# Improvement of central vein ultrasound-guided puncture success using a homemade needle guide—a simulation study

**DOI:** 10.1186/s13054-023-04661-w

**Published:** 2023-09-30

**Authors:** Antoine Villa, Vladimir Hermand, Vincent Bonny, Gabriel Preda, Tomas Urbina, Maxime Gasperment, Paul Gabarre, Louai Missri, Jean-Luc Baudel, Daniel Zafimahazo, Jérémie Joffre, Hafid Ait-Oufella, Eric Maury

**Affiliations:** 1 Medical Intensive Care Unit, Hôpital Saint Antoine, Assistance Publique-Hôpitaux de Paris (APHP), Saint-Antoine University Hospital, Sorbonne University, 75012 Paris, France; 2 Learning Planet Institute, 75004 Paris, France; 3 Faculty of Medicine, Sorbonne University, 75013 Paris, France; 4 INSERM UMR_S938, Centre de Recherche Saint-Antoine (CRSA), 75571 Paris Cedex 12, France; 5 Paris Cardiovascular Research Center, INSERM U970, Paris University, Paris, France; 6 Pierre Louis Institute of Epidemiology and Public Health, INSERM U1136, Sorbonne University, Paris, France

**Keywords:** Catheterization, Central venous catheter insertion, Ultrasonography

## Abstract

**Background:**

Out-of-plane (OOP) approach is frequently used for ultrasound-guided insertion of central venous catheter (CVC) owing to its simplicity but does not avoid mechanical complication. In-plane (IP) approach might improve safety of insertion; however, it is less easy to master. We assessed, a homemade needle guide device aimed to improve CVC insertion using IP approach.

**Method:**

We evaluated in a randomized simulation trial, the impact of a homemade needle guide on internal jugular, subclavian and femoral vein puncture, using three approaches: out-of-plane free hand (OOP-FH), in-plane free hand (IP-FH), and in-plane needle guided (IP-NG). Success at first pass, the number of needle redirections and arterial punctures was recorded. Time elapsed (i) from skin contact to first skin puncture, (ii) from skin puncture to successful venous puncture and (iii) from skin contact to venous return were measured.

**Results:**

Thirty operators performed 270 punctures. IP-NG approach resulted in high success rate at first pass (jugular: 80%, subclavian: 95% and femoral: 100%) which was higher than success rate observed with OOP-FH and IP-FH regardless of the site (*p* = .01). Compared to IP-FH and OOP-FH, the IP-NG approach decreased the number of needle redirections at each site (*p* = .009) and arterial punctures (*p* = .001). Compared to IP-FH, the IP-NG approach decreased the total procedure duration for puncture at each site.

**Conclusion:**

In this simulation study, IP approach using a homemade needle guide for ultrasound-guided central vein puncture improved success rate at first pass, reduced the number of punctures/redirections and shortened the procedure duration compared to OOP and IP free-hand approaches.

**Supplementary Information:**

The online version contains supplementary material available at 10.1186/s13054-023-04661-w.

## Introduction

Current guidelines recommend ultrasound guidance for central venous catheter (CVC) insertion [1, 2] which, compared to landmark strategy, increases success cannulation rate and decreases the number of insertion attempts, time to cannulation, and complications such as arterial punctures or pneumothorax [3–5].

For optimal safety, ultrasound-guided CVC insertion requires to visualize the vessel and the needle. Two approaches are available to visualize the needle: the out-of-plane (OOP) approach and the in-plane (IP) approach [6, 7] which are defined according to needle's position relative to the ultrasound beam. OOP is widely utilized, and while safer compared to landmark, it still has potential for complications. During OOP approach, the needle is perpendicular to the ultrasound beam and appears as a spot that represents its intersection with the ultrasound beam. With OOP approach, the operator does not continuously visualize the needle tip and faces risks of arterial injury or multiple sticks due to the need for redirection.

Conversely, with IP approach, the needle is within the plane of the ultrasound beam and is tracked from skin's perforation to vessel penetration. As a result, IP approach might enhance safety by minimizing the risk of injury to adjacent structures. However, IP approach requires accurate needle alignment with the ultrasound beam making this approach highly demanding. Needle guiding devices or methods to facilitate IP approach are available but tend to be expensive (several hundred €), sometimes specific to particular probes, and remain poorly investigated so far [8–13].

### Methods

We designed an open-source 3D printed needle guide to facilitate IP-CVC insertion. The 7.5-MHz linear probe HFL38 available on the M-Turbo® device (Sonosite, Bothewell, MA) was scanned with an EinScan Pro 2X device. Scanning process required two hours per model. We obtained the 3D mold of the probe. The needle guide made of poly lactic acid was wrapped around the digitalized probe using Autodesk Fusion 360. A dedicated needle-guiding railway was incorporated into the mold along the narrow side of the probe. The railway must allow adjustments in needle angulation, while also maintaining tightly the needle in the ultrasound beam. Finally, the needle guide was 3D printed on a Prusa I3mkiiiS printer (Prusa Research, Praha, CZ). Six needle guide iterations were tested before obtaining the perfect fit for the probe (Additional file 2: Figure S1). Design and conception took 26 h for a cost of 1€. The process patent approval is pending.

We conducted a prospective, randomized study among residents and board-certified physicians from either ICU or emergency department to assess the needle guide in the setting of central venous puncture performed on inanimate manikin. All the participants had received a training in ultrasound-guided CVC insertion, but their proficiency was inhomogeneous. The participants were classified according on whether they had performed more or less than twenty CVCs insertion on patients. All participants were given a one-hour US-CVC lecture about US-guided IP and OOP approaches on simulator.

Two simulators were used i) (the Blue Phantom II, CAE Healthcare St. Louis, MO) which allows internal jugular and subclavian puncture and ii) (the Gen II Femoral Vascular Access Ultrasound Training Model, CAE Healthcare St. Louis, MO) which permits femoral vein puncture. Blue fluid return confirmed venous puncture, whereas return of red fluid ruled in arterial puncture. Before the study, the participants were given a 10-min session to the needle guide and the simulators. Each operator performed needle puncture of the jugular, subclavian and femoral veins on the simulators, with three different techniques, assigned in random order: out-of-plane free hand (OP-FH), in-plane free hand (IP-FH) and in-plane with needle guide (IP-NG) (Additional file 2: Figure S2). The procedure was limited to venous puncture and did not include guide wire insertion.

We recorded: success rate at first pass, number of needle redirections (and skin breaches) and duration of different parts of the puncture procedure (i) from skin contact to first skin puncture, (ii) from first skin puncture to successful venous puncture and (iii) from skin contact to successful venous return (entire puncture procedure). Uncomplicated puncture was defined as puncture (with venous return) performed in less than 120 s without arterial puncture.

Data are expressed as median [1st and 3rd interquartile]. Success rates are compared using Chi-2 or Fisher exact test as required. The number of needle re-directions and the procedure time are compared using bilateral Wilcoxon matched pairs test. Significance is set for p less than 0.05.

## Results

Thirty operators (age: 30 [27–37] years, female 20%) being board-certified physicians (40%) or residents (60%) agreed to participate in the study. Twenty-four of the participants belonged to the ICU team, while the six remaining were physicians working in the emergency department. Sixteen operators (53%) reported clinical proficiency of more than 20 CVC insertions. All participants performed the nine scheduled catheter insertions for a total of 270 punctures.

**Fig. 1:**
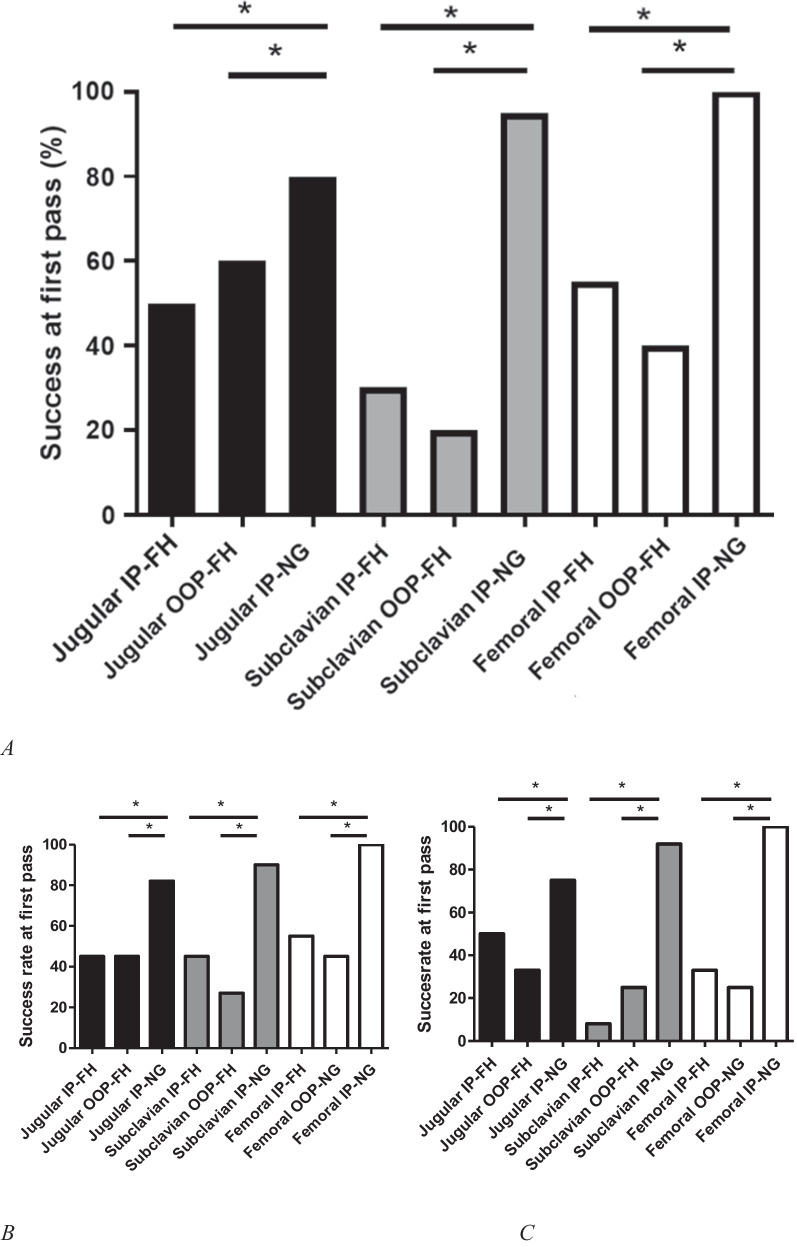
Success rate at first pass according to the site where the puncture was performed, and the approach used in all operators (**A**) and among operators having performed more than 20 CVC insertions (**B**) or less than 20 CVC insertions (**C**). Success was defined by syringe filling by blue liquid. IP-FH: in-plane free hand, IP-NG: in-plane needle guided, OOP-FH: out-of-plane free hand. *p < 0.05 Chi-2 test.

Utilizing NG-IP resulted in a high success rate at first pass (jugular: 80%, subclavian: 95% and femoral 100%) and was significantly higher than success rate at first pass observed with OOP-FH and IP-FH approaches whatever site (*p* < 0.001) (Fig. 1). Analyzing data according to CVC insertion experience revealed similar trends (Fig. 1).

Compared to OOP-FH and IP-FH, IP-NG approach decreased the number of needle punctures (skin breaches) or redirections at all sites (*p* = 0.009) (Table 1). IP-NG use was associated with less arterial puncture than free-hand approaches (2/90 vs 30/180, *p* = 0.002). Uncomplicated puncture occurred more frequently with NG-IP compared to IP-FH at subclavian site.

**Table 1:** Needle redirections and needle passes (skin breaches), arterial puncture and uncomplicated puncture according to the different approaches at jugular, subclavian and femoral site

Site	Approach	Needle redirection/needles passes n	Arterial puncture n (%)	Uncomplicated puncture %
Jugular	IP-FH	2 [1–3]	1 (3%)	97
	OOP-FH	2 [1–2]	2 (7%)	93
	IP-NG	1 [1–1]*^£^	1 (3%)	93
Subclavian	IP-FH	3 [1–5]	6 (17%)	76
	OOP-FH	3 [1–4]	10 (33%)	83
	IP-NG	1 [1–1]*^£^	1 (3%)^£^	100*
Femoral	IP-FH	1 [1–3]	3 (10%)	93
	OOP-FH	2 [1–2]	8 (27%)	93
	IP-NG	1 [1–1]*^£^	0 (0%) £	97

Needle redirections and needle passes (skin breaches) are given for each puncture (median and IQR). Arterial puncture are defined by reflux of red fluid in the syringe reported at least once for a puncture (percentage). Uncomplicated puncture are defined as successful puncture completed in < 120 s without arterial puncture (percentage) according to the different approaches at jugular, subclavian and femoral site

*IP-FH* in-plane free hand, *IP-NG* in-plane needle guided, *OOP-FH* out-of-plane free hand

*p < 0.05 IP-FH versus IP-NG

^£^*p* < 0.05 IP-NG versus OOP-FH

Compared to IP-FH approach, IP-NG approach decreased the time elapsed between skin probe application and first needle puncture at each site (Additional file 2: Table S1). Time from skin puncture to successful venous fluid return was shorter with the needle guide compared to the free-hand techniques for subclavian and femoral punctures. Finally, compared to IP-FH approach, IP-NG approach decreased the duration of the total procedure from skin probe contact to successful venous return at all sites (Additional file 2: Table S1).

The duration of the total procedure using IP-NG approach was significantly lower compared to OOP-FH approach at subclavian and femoral sites (Additional file 2: Table S1).

IP-NG use resulted in significantly shorter total procedure duration compared to both IP-FH and OOP-FH at subclavian site whatever proficiency (18[13–33] vs 34 [17–133] and 33[18–114] *p* < 0.001, for operators having inserted > 20 CVC) and (25[21–45] vs 73 [38–94] and 50 [28–119] *p* < 0.001, for operators having inserted < 20 CVC).

## Discussion

Whereas guidelines actually recommend ultrasound use for CVC insertion, some uncertainty remains for the best approach to use between OOP and IP [1, 7, 14].

Out-of-plane approach is most often used for jugular and femoral CVC insertion owing to its convenience. However, it is associated with risk of injury of posterior venous wall [15, 16] and of adjacent structures during needle progression [16, 17]. This is why we designed a device aimed at making IP procedures easier with minimal risk of complications.

We demonstrate here that, compared to IP-FH and OOP-FH, IP-NG approach significantly increases success rate at first pass in all sites. This is in keeping with previous studies reporting similar success rate with guiding system for jugular (82% and 81%) [18, 19] and subclavian puncture [20] even among inexperienced operators [19].

Accidental arterial puncture with the NG-IP approach was observed only once at the jugular and subclavian site and never at the femoral site. Using a different needle guiding system, Augoustides et al. reported a rate of accidental arterial puncture at jugular site of 10%, similar to the rate observed with the landmark approach [16]. This could be due to the guiding device used in this study (based on OOP approach), which allows needle visibility only at the depth of the vein. The device we use in the present study places the needle in the ultrasound beam and visualizes the whole path of the needle. This design associated with the possibility to modify the angulation of the needle permits puncture whatever the depth of the vessel [20] without requiring supplemental accessories [16, 20].

The study reported here presents anyway several limits, the most important being that we should confirm our data in the clinical setting. Moreover the device was not used under a sterile sheath. This has not been investigated in the present study. The prototype had first to be assessed in a simulation model before being tested in the real life.

We chose to include operators with limited proficiency considering that whether guide improves performance this would be true especially among operators with limited experience. Compared to IP-FH and OOP-FH, IP-NG translated in shorter total procedure duration only for subclavian puncture whatever proficiency. It should also be outlined that all IP-NG subclavian punctures (considered as the more complex puncture) performed by operators with the smallest experience were completed in less than 48 s. This confirms that needle-guiding interest is relevant especially for subclavian puncture [9].

This study presents the first ultrasound needle-guiding device for IP (CVC) insertion, constructed using an open-source and homemade method. The cost of production and ease of construction are noteworthy. This approach could allow every operator to obtain a guiding device that is adapted to his ultrasound probes.

The online version contains supplementary material

### Supplementary Information

**Additional file 1.** Needle Guide mp4 showing subclavian puncture using successively Out-of-Plane Free Hand, In Plan-Free Hand and In-Plane Needle Guided approaches. Subclavian vein appears on the ultrasonography screen as a sausage shape when In-Plane approach is used. In-Plane Needle Guided approach permits immediate visualization of the needle and a successful puncture at first pass.

**Additional file 2. Figure S1** describing needle guide designing with Panel A showing: the scanning of 7.5 MHz linear probe HFL38 with an EinScan Pro 2X. Panel B showing: the adjunction of a railway dedicated to guide the needle on the little side of the mold probe. Panel C showing: the printed guide fitting to the probe and the adaptation of the railway, which should be wide enough to permit angulation of the needle and thigh enough to maintaining the needle in the ultrasound beam in all angulations. Panel D: the different models printed among which the final prototype was chosen. **Figure S2** showing puncture of blue phantom (and the corresponding echo images) using the three approaches (IP-FH upper panel, OOP-FH middle panel and IP-NG lower panel). **Table S1**: Time in seconds (median and IQR) of different components of procedure elapsed 1) from probe contact with skin to first puncture, 2) from first puncture to successful venous return, and of 3) from skin contact to venous return (whole procedure). IP-FH: In-plane Free Hand, IP-NG: In-plane Needle Guided, OOP-FH Out-of-plane Free Hand.* p<.05 IP-FH vs IP-NG, £ p<.05 IP-NG vs OOP-FH. Needle Guide mp4 showing subclavian puncture using successively Out-of-Plane Free Hand, In Plan-Free Hand and In-Plane Needle Guided approaches. Subclavian vein appears on the ultrasonography screen as a sausage shape when In-Plane approach is used. In-Plane Needle Guided approach permits immediate visualization of the needle and a successful puncture at first pass.

## Data Availability

Datasets were collected on an Xcel file are stored by AV and EM and can be accessed upon request.
