# Supplementary material for: Improvement of central vein ultrasound-guided puncture success using a homemade needle guide—a simulation study
Source: Crit Care. 2023 Sep 30;27:379. doi: 10.1186/s13054-023-04661-w (PMC10543855; doi:10.1186/s13054-023-04661-w)
Supplement: Supplementary file 2 — Additional file 2. Figure S1 describing needle guide designing with Panel A showing: the scanning of 7.5 MHz linear probe HFL38 with an EinScan Pro 2X. Panel B showing: the adjunction of a railway dedicated to guide the needle on the little side of the mold probe. Panel C showing: the printed guide fitting to the probe and the adaptation of the railway, which should be wide enough to permit angulation of the needle and thigh enough to maintaining the needle in the ultrasound beam in all angulations. Panel D: the different models printed among which the final prototype was chosen. Figure S2 showing puncture of blue phantom (and the corresponding echo images) using the three approaches (IP-FH upper panel, OOP-FH middle panel and IP-NG lower panel). Table S1: Time in seconds (median and IQR) of different components of procedure elapsed 1) from probe contact with skin to first puncture, 2) from first puncture to successful venous return, and of 3) from skin contact to venous return (whole procedure). IP-FH: In-plane Free Hand, IP-NG: In-plane Needle Guided, OOP-FH Out-of-plane Free Hand.* p<.05 IP-FH vs IP-NG, £ p<.05 IP-NG vs OOP-FH. Needle Guide mp4 showing subclavian puncture using successively Out-of-Plane Free Hand, In Plan-Free Hand and In-Plane Needle Guided approaches. Subclavian vein appears on the ultrasonography screen as a sausage shape when In-Plane approach is used. In-Plane Needle Guided approach permits immediate visualization of the needle and a successful puncture at first pass. [file 13054_2023_4661_MOESM2_ESM.docx]

**Supplemental materials**


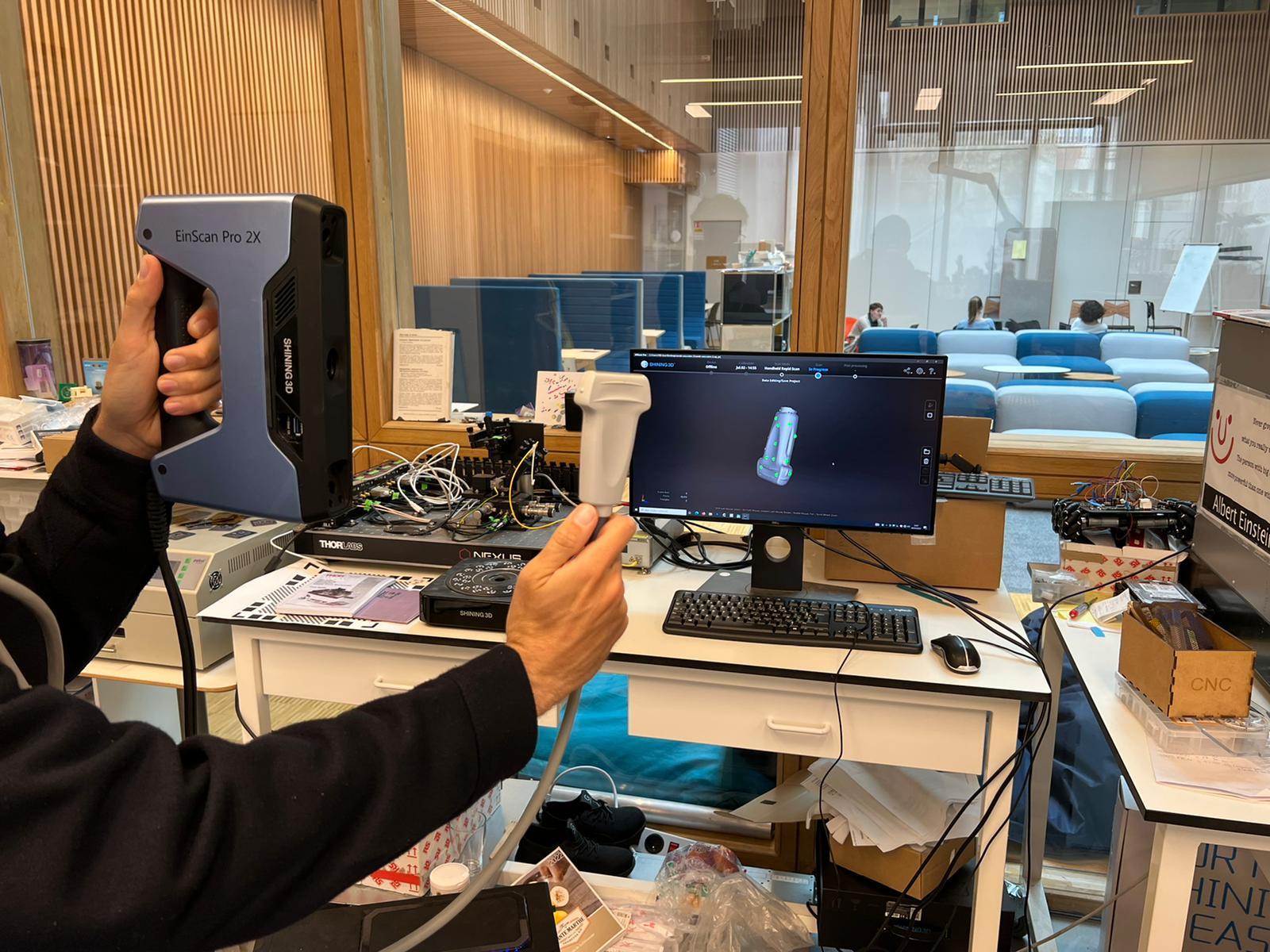

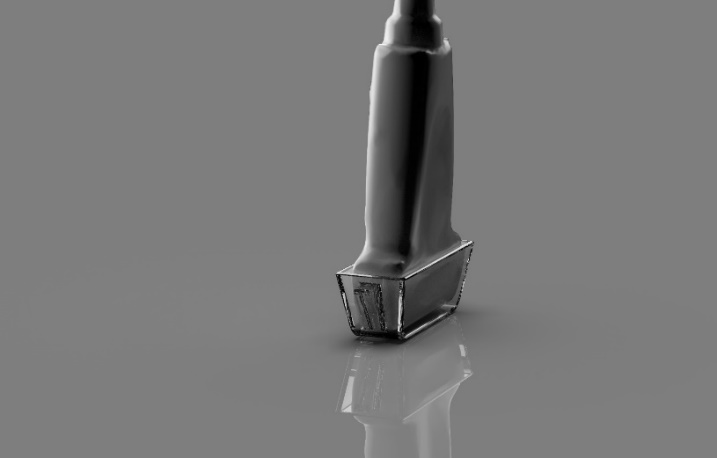

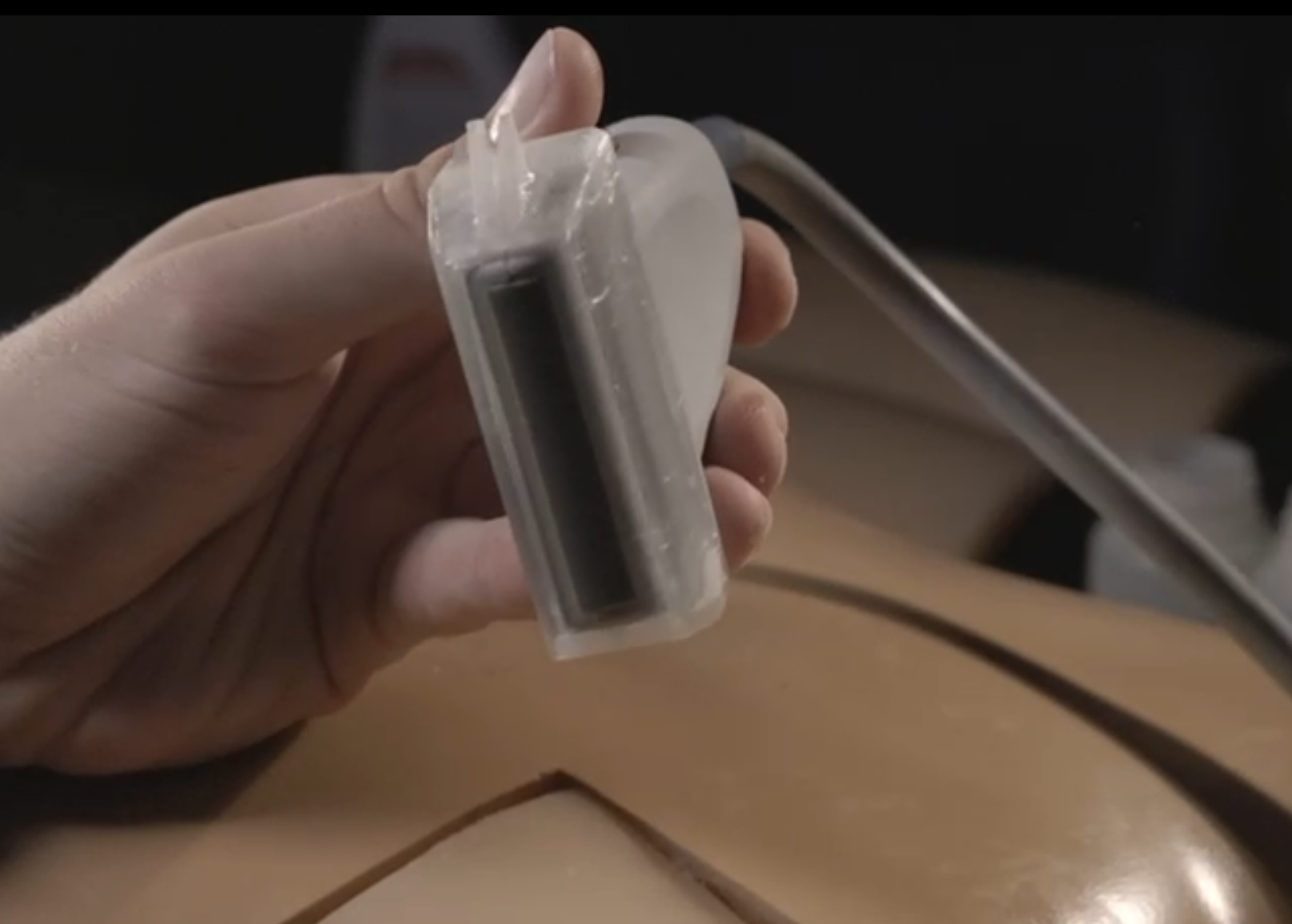


C

B

A


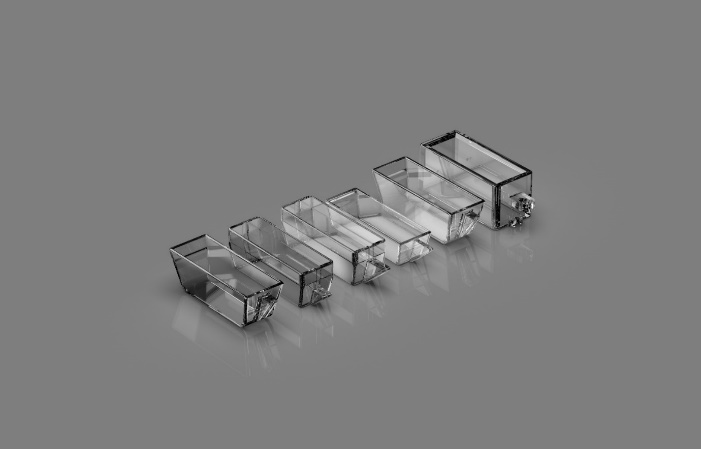


D

**Figure S1** describing needle guide designing with **Panel A** showing**:** the scanning of 7.5 MHz linear probe HFL38 with an EinScan Pro 2X.  **Panel B** showing**:** the adjunction of a railway dedicated to guide the needle on the little side of the mold probe. **Panel C** showing: the printed guide fitting to the probe and the adaptation of the railway, which should be wide enough to permit angulation of the needle and thigh enough to maintaining the needle in the ultrasound beam in all angulations. **Panel D:** the different models printed among which the final prototype was chosen.


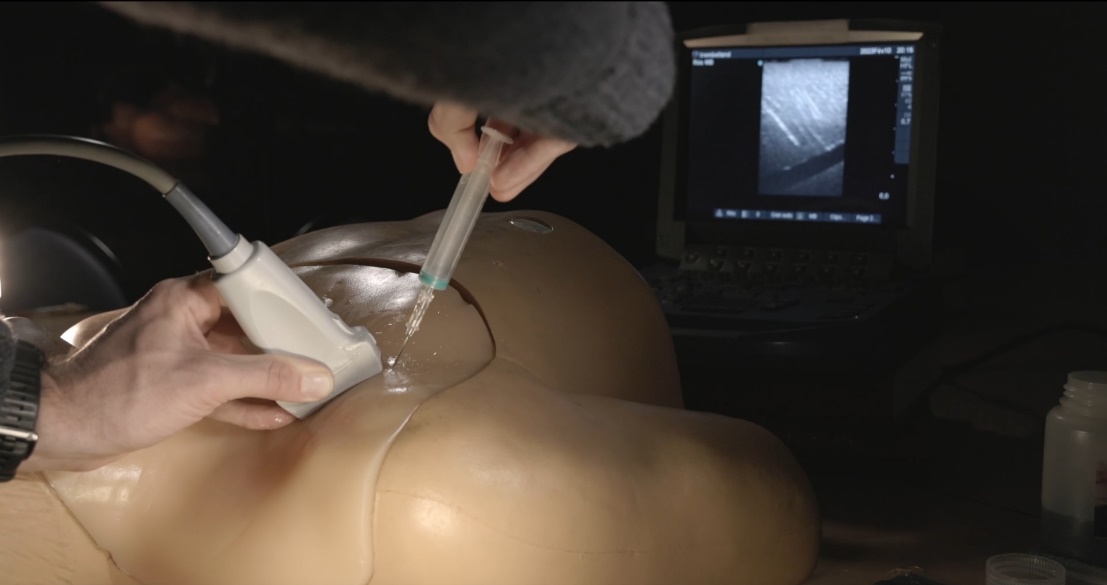

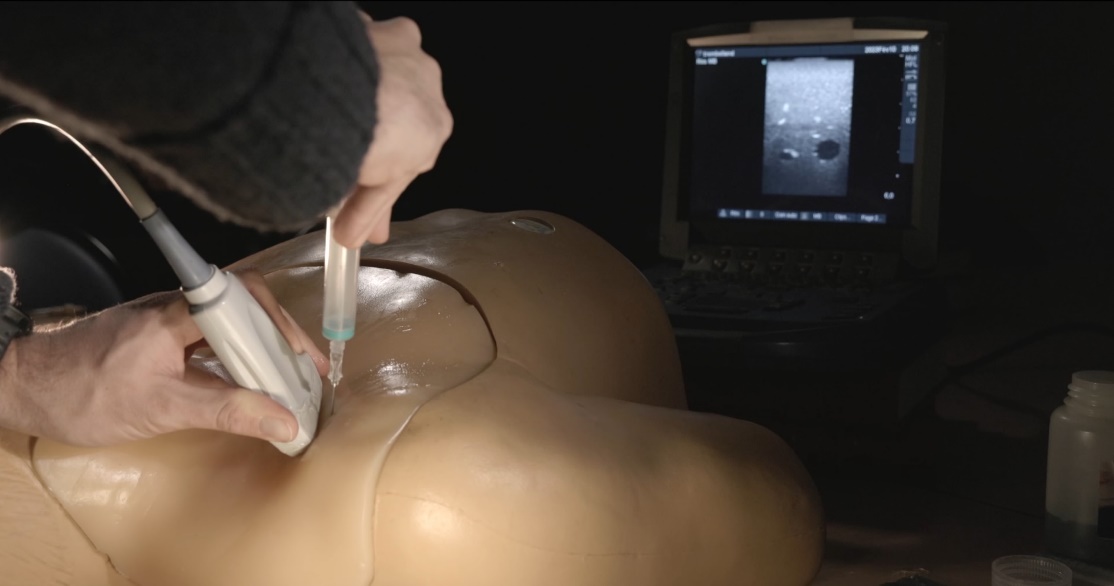

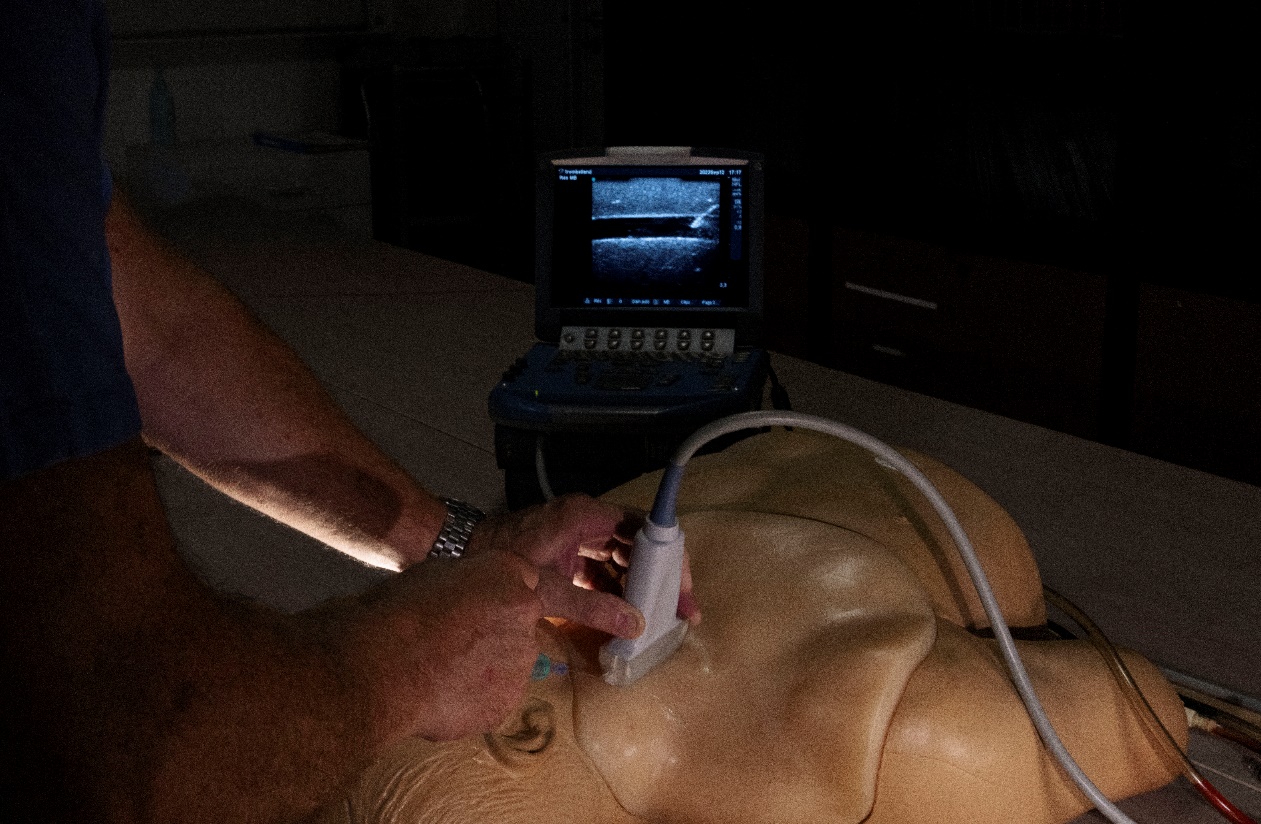


**Figure S2** showing puncture of blue phantom (and the corresponding echo images) using the three approaches (IP-FH upper panel, OOP-FH middle panel and IP-NG lower panel).

| ***Site*** | ***Approach*** | **From skin contact to**  **puncture** | **From puncture to venous return** | **Whole procedure** |
| --- | --- | --- | --- | --- |
| ***Jugular*** | *IP-FH* | 23 [10-36] | 9 [4-18] | 31 [16-66] |
|  | *OOP-FH* | 12 [6-19] | 7 [3-16] | 17[12-28] |
|  | *IP-NG* | 11 [9-29] *£ | 6 [4-8]*£ | 19 [13-35]*£ |
| ***Subclavian*** | *IP-FH* | 21 [12-43] | 29 [11-66] | 52 [29-120] |
|  | *OOP-FH* | 17 [10-26] | 27 [9-66] | 42 [20-116] |
|  | *IP-NG* | 15 [8-21]* £ | 10 [6-13] *£ | 23 [18-37]*£ |
| ***Femoral*** | *IP-FH* | 16 [10-39] | 10 [6-30] | 30 [21-57] |
|  | *OOP-FH* | 10 [7-18] | 12 [6-22] | 26 [16-33] |
|  | *IP-NG* | 12[6-18]*£ | 7 [5-10]*£ | 20 [13-26]*£ |

**Table S1 :** Time in seconds (median and IQR) of different components of procedure elapsed 1) from probe contact with skin to first puncture, 2) from first puncture to successful venous return, and of 3) from skin contact to venous return (whole procedure). IP-FH: In-plane Free Hand, IP-NG: In-plane Needle Guided, OOP-FH Out-of-plane Free Hand.* p<.05 IP-FH vs IP-NG, £ p<.05 IP-NG vs OOP-FH

**Needle Guide mp4** showing subclavian puncture using successively Out-of-Plane Free Hand, In Plan-Free Hand and In-Plane Needle Guided approaches. Subclavian vein appears on the ultrasonography screen as a sausage shape when In-Plane approach is used. In-Plane Needle Guided approach permits immediate visualization of the needle and a successful puncture at first pass.
